# Correlation analysis on physicochemical and structural properties of sorghum starch

**DOI:** 10.3389/fnut.2022.1101868

**Published:** 2023-01-10

**Authors:** Shouxin Yan, Zhao Li, Bin Wang, Tingting Li, Zhiyang Li, Nan Zhang, Bo Cui

**Affiliations:** ^1^State Key Laboratory of Biobased Material and Green Papermaking, Qilu University of Technology, Shandong Academy of Sciences, Jinan, China; ^2^School of Food Science and Engineering, Qilu University of Technology, Shandong Academy of Sciences, Jinan, Shandong, China

**Keywords:** sorghum starch, amylose content, pasting properties, thermal properties, correlation analysis

## Abstract

This manuscript analyzed physicochemical and structural properties of 30 different types of sorghum starches based on their apparent amylose content (AAC). Current results confirmed that sorghum starch exhibited irregular spherical or polygonal granule shape with 14.5 μm average particle size. The AAC of sorghum starch ranged from 7.42 to 36.44% corresponding to relative crystallinities of 20.5 to 32.4%. The properties of enthalpy of gelatinization (ΔH), peak viscosity (PV), relative crystallinity (RC), degree of double helix (DD), degree of order (DO), and swelling power (SP) were negatively correlated with AAC, while the cool paste viscosity (CPV) and setback (SB) were positively correlated with AAC. Correlations analyzed was conducted on various physicochemical parameters. Using principal component analysis (PCA) with 20 variables, the difference between 30 different types of sorghum starch was displayed. Results of current study can be used to guide the selection and breeding of sorghum varieties and its application in food and non-food industries.

## 1. Introduction

Sorghum [*Sorghum bicolor* (L.) Moench] is a significant cereal crop which ranks the fifth largest cereal crop in the world after barley, corn, wheat and rice (1). Due to its high drought tolerance, sorghum is widely cultivated in Africa and Asia, even in the areas of salinity. Under the same water condition, sorghum has higher water-use-efficiency compared to other types of crops, such as maize, this makes it becomes a primary source of food in the world (2, 3). The other advantage of sorghum is its high antioxidant activity, which is demonstrated by the high phenolic compounds in its seed coat. And these phenolic compounds contribute to the abundant pigments in the coat of sorghum seed (4, 5). As sorghum is rich in tannins and others substances, it become the main raw material for favor liquor (6). Besides its wide application in liquor-making industry and sorghum food due to healthy substances such as nutrition, antioxidant, antiobesity, antidiabetic, antibacterial, and anticancer activities (7). Due to the above advantages, sorghum has a wide range of applications in food, feed, liquor manufacturing, hypoglycemic medicines, and minerals (7, 8).

According to previous studies on sorghum properties, starch is the primary component in sorghum grain, which obtains a mass fraction from 65.3 to 81.0% with average value of 79.5% (5). Currently, there are a growing interest in the physicochemical properties of different sorghum starch. The physicochemical properties of sorghum starch determine its application. Sorghum starch is rich in slow-digesting and resistant starch, which are often used to make hypoglycemic and anti-obesity foods due to its poorly digested property. Sorghum starch is the most important carbon source in white wine fermentation, not only provides energy for the wine bent, but also is a raw material for esters, alcohols and acid aromatics. And the fine structure of starch largely affects the efficiency of white wine fermentation and product quality (9). The pasting properties of sorghum starch makes it can be used as thickener and adhesive (10). It is clear that the researches on the physicochemical properties of sorghum starch are important to promote its further application.

The size of sorghum starch ranges from 10 to 16 μm, and it is mainly found in the endosperm (11). Starch consists of two types of polysaccharide molecules: amylose (linear) and amylopectin (branched). In previous studies, Chen et al. (12) stated the amylose content of sorghum starch ranged from 0.18 to 28.21%. Their study also pointed out the ratio of amylose and amylopectin might affect physicochemical, thermal, and rheological characteristics of starch. Sorghum starches showed the typical A-type crystallinity polymorph, with strong diffraction peaks at 2θ around 15.4, 17.2, 18.2, and 23.2°, and another weaker peak at 2θ around 20° (13). The crystalline structures of starch granules are affected by the amylose-amylopectin ratio, degree of branching, and chain length of the amylopectin (14). Sang et al. (15) found that a high amylose content in sorghum starch might result in low peak viscosity, peak pasting temperature and pasting enthalpy, which revealed that amylose content might negatively impact viscosity and thermal properties. And it is also negatively correlated with the water solubility index and relative crystallinity (16). Singh et al. (17) investigated the amylose content in Indian sorghum starch ranged from which ranging from 11.2 to 28.5%. Boudries et al. (18) compared the physicochemical, functional, and structural properties of starch isolated from red and white Algerian sorghum. And they discovered that the amylose content of sorghum starch was slightly different from corn and wheat starch, but sorghum starch exhibited higher water holding capacity and solubility than those of wheat starch.

Currently, most research focused on maize starch, wheat starch, potato starch, and quinoa starch. To the best of our knowledge, research on the physicochemical properties of sorghum starch analysis used only a limited number of samples, which emphasized on comparing different varieties sorghum starch (less than 15 samples) and starch from other plant sources (17, 18). The sorghum in this study was provided by the Chinese Academy of Sciences, which were grown in Gansu, Beijing, and Dongying with red, yellow, and white appearance. In this article, sorghum species expanded sorghum species to thirty varieties that were all cultivated in China, which will fill up the blank in the starch database. By analyzing the amylose contents in different sorghum starch, the relationship between sorghum varieties and characteristics in physicochemical and structural levels will be explored. This research will contribute to the selection and breeding of sorghum varieties for starch extraction by allowing for the targeted selection of preferred sorghum types, and give food industry a good selection of sorghum starch and develop new product in market.

## 2. Materials and methods

### 2.1. Materials

Thirty varieties of sorghum were bred by the Chinese Academy of Sciences and were in the Academy’s sorghum database (Table 1). Maize amylopectin and potato amylose were purchased from Sigma–Aldrich Chemical, Co. (Shanghai, China). Iodine and potassium iodide were purchased from Macklin Biochemical Co., Ltd (Shanghai, China). Sodium hydroxide, urea and dimethyl sulfoxide were purchased from Sinopharm Chemical Reagents Co., Ltd (Shanghai, China).

**TABLE 1:** Product information of sorghum samples.

ID in database	Sample code	Origin	Color
PI641807	S1	Gansu	White
PI586454	S2	Gansu	Red
PI220636	S3	Beijing	Red
00013897	S4	Beijing	Yellow
PI30204	S5	Gansu	Red
PI152968	S6	Dongying	Yellow
PI201723	S7	Gansu	Yellow
PI287595	S8	Beijing	Red
PI287632	S9	Dongying	Yellow
PI152743	S10	Dongying	Red
PI287589	S11	Gansu	Red
PI287590	S12	Gansu	White
PI287596	S13	Gansu	Yellow
PI651495	S14	Gansu	Yellow
PI454574	S15	Gansu	Red
PI669332	S16	Gansu	Red
PI570998	S17	Gansu	Yellow
PI570987	S18	Gansu	Red
PI641828	S19	Dongying	Red
PI217892	S20	Dongying	Yellow
PI177156	S21	Gansu	Red
PI655983	S22	Gansu	White
PI563094	S23	Gansu	White
GW000019	S24	Gansu	Yellow
PI570998	S25	Dongying	Red
PI570955	S26	Dongying	White
PI571134	S27	Dongying	Yellow
00004640	S28	Gansu	Red
00004789	S29	Dongying	Red
PI260210	S30	Beijing	Yellow

The starch ID was provided by the Chinese Academy of Sciences.

### 2.2. Starch isolation and purification

Thirty different kinds of sorghum were used to extract their starches following a previous method (19) with some modifications. The sorghum grain was immersed in a 0.25% (w/v) NaOH solution at 1:5 (w/v) ratio for 24 h. It was cleaned and crushed for 10 min using a colloid mill (YL90S-2, Zhejiang, China) followed by passing through a 200 mesh screen. Then, sodium hydroxide (0.25% w/v) was added to keep the soaking going for another 24 h. Afterward, the slurry was centrifuged for 20 min at 5,000 rpm. The precipitate was resuspended and centrifuged, and this procedure was repeated with anhydrous ethanol firstly and distilled water for three times. Finally, the starch was dried at 40°C in oven for 24 h in purpose of future analysis.

### 2.3. Moisture content (MC)

The moisture content of starch was determined using a halogen moisture analyzer (MA45C, Germany) following the method of Li et al. (20). Sorghum starch (1 g) was weighed and distributed in an aluminum foil dish. Samples were then baked at 110°C until the constant weight was obtained. The results were expressed as a percentage of total weight basis. Each measurement was performed in triplicate.

### 2.4. Apparent amylose content

The AAC was ascertained using the iodine binding-based method (21, 22) with minor modifications. KI-I_2_ solution was prepared by dissolving 0.5 g KI and 0.05 g I_2_ in 25 ml distilled water, and the KI-I_2_ solution was kept in dark at 4°C. Maize amylopectin and potato amylose were used to prepare starch standard curve with amylose content of 0, 20, 40, 60, 80, and 100%. Eight milliliters of urea-dimethyl sulfoxide solution (1:9 v/v) was added to 20 mg of starch, following by mixing using vortex for 2 min. The mixture was incubated in an 85°C water bath for 30 min and shaked every 5 min. The starch solution was cooled down to room temperature and fixed into a 25 ml volumetric flask. Aliquots (3 ml) of starch solution was mixed with 1 mL of KI-I_2_ solution to make 50 ml samples solution with distilled water. The sample was allowed to stand at 25°C for 15 min before measuring absorbance at 620 nm. The Amylose content was calculated based on the regression Equation 1. *x* is absorbance, y is the amylose content.


(1) y=0.0133⁢x+0.0413

### 2.5. Scanning electron microscopy

Scanning electron microscopy (SEM; Hitachi SU3500, Tokyo, Japan) was used to examine sorghum starch at a 5 kV accelerated voltage (23). The samples were gold plated after being coated with double-sided carbon coated tape. The microstructure of sorghum starch was then imaged using SEM.

### 2.6. Swelling power and water solubility index

Swelling power (SP, g/g) and water solubility index (WSI, %) were determined using a method modified from Tsai et al. (24). Starch (0.15 g) was accurate weighted (±0.1 mg) and mixed with 10 mL of distilled water in a 15 ml centrifuge tube, and the sample was prepared in quintuplicate. The tubes were heated in a water bath at 55, 65, 75, 85, and 95°C, respectively. The incubation was carried out for 1 h with frequent shaking at first 10 min. The tubes were then centrifuged at 3,000 × *g* for 20 min after cooling down through room temperature water to 25°C. After transferring the supernatant to a petri dish, then supernatant was dried and weighed (W1). And the sediment was weighed and recorded as Ws. The following formulas were used to calculate WSI and SP:


(2) $$W\,S\,I=\frac{W\,1}{W\,0}\times 100\%$$

(3) $$S\,P\,\left(g/g\right)=\frac{W\,s}{W\,0\times \left(1-W\,S\,I\right)}$$

### 2.7. ATR-FTIR spectroscopy analysis

The short-range ordered structures of different sorghum starches were evaluated by ATR-FTIR spectroscopy (Bruker, V70FTIR, Berlin, Germany). The sample was set flat on the diamond ATR crystal’s surface in a homogeneous manner, the air background was gathered. The sample was subsequently scanned. ATR-FTIR spectroscopy in the range of 1,100–900 cm^–1^ was deconvoluted using OMNIC 9.2 software to determine the absorbance of 1,047, 1,022, and 995 cm^–1^. Then, the values of 1,047/1,022 cm^–1^ (DO) and 995/1,022 cm^–1^ (DD) were calculated.

### 2.8. X-ray diffraction

The crystalline characteristics of sorghum starch were obtained using an X-ray diffractometer (SmartLabSE, Rigaku, Japan). The parameters were tested using 40kV and 30mA with Cu kα radiation. The samples, with a 0.03 step size, were measured between 5° and 40° (25). The relative crystallinity (RC) of the sample was calculated using JADE 6.5 software based on Kang et al. (26).


(4) $$RC\left(\%\right)=\frac{C\,r\,y\,s\,t\,a\,l\,a\,r\,e\,a}{A\,m\,o\,r\,p\,h\,o\,u\,s\,a\,r\,e\,a+C\,r\,a\,s\,t\,a\,l\,a\,r\,e\,a}$$

### 2.9. Pasting analysis

Pasting properties of the samples were determined using a rapid visco-analyzer (RVA, Perten Instruments, Australia) based on to the method reported by Ambigaipalan et al. (27). The samples (2.5 g) were disseminated in distilled water in an aluminum tube (25 ml). The initial agitation speed was 960 rpm for 10 s to ensure consistency of the diffusion, and the speed was then slowed down to 160 rpm. The starch suspension was held at 50°C for 1 min, then heated up to 95°C at 12°C/min and kept at 95°C for 2.5 min. Afterwards, the suspension was reduced to 50°C at the same rate and held for 2 min. Pasting parameters were determined, including pasting temperature (PT), peak temperature (PKT), peak viscosity (PV), hot paste viscosity (HPV), cool paste viscosity (CPV), breakdown (BD = PV − HPV), setback (SB = CPV − HPV), stability ratio (SR = 100 × HPV/PV) and setback ratio (BR = CPV/HPV).

### 2.10. Differential scanning calorimetry (DSC)

The melting curve of sorghum starch was determined using a NETZSCH DSC analyzer (DSC- 200fc, NETZSCH, Selb, Germany) based on to the method reported by Yang et al. (28). Starch samples (2 mg) were mixed with 6 μl of ultrapure water in a DSC crucible. The airtight crucible was then equilibrated at room temperature for 12 h. The samples were heated from 30 to 120°C at a heating speed of 10°C/min. An empty crucible was used as control. The onset (*T*o), peak (*T*p), conclusion (*T*c) temperatures of gelatinization, and the enthalpy change (Δ*H*), were recorded.

### 2.11. Statistical analysis

The mean and standard deviation were obtained by SPSS V. 25 software, and the significance of the results was evaluated by analysis of variance. At *p <* 0.05, significant differences in the mean values were established. The correlation analysis between starch properties was determined by SPSS software V. 25. The principal component analysis (PCA) results were obtained in Origin 2021 V. 9.8 software.

## 3. Results and discussion

### 3.1. Moisture content (MC) and apparent amylose content (AAC)

The moisture content of thirty sorghum starches ranged from 8.4 ± 0.3% in S19 to 13.5 ± 0.3% in S18 (Table 2). Starch with water content between 9 to 12% accounted for 60% of the total starch species in this study. The moisture content of sorghum starch in Belhadi et al. (29) ranged from 8.44 to 11.39%, which was close to the results of this study. AAC varied from 7.42 ± 0.21% in S4 to 36.44 ± 1.06% in S29 (Table 2), with a mean value of 25.58%. The AAC determined by iodometric method was reported between 5.18 and 28.5% (17, 18, 30, 31). Chen et al. (12) stated the amylose content in sorghum starch determined by the iodometric method ranged from 0.18 to 28.21%, from which a low value of AAC was obtained. It could be attributed to the different varieties of starch. The amounts of iodine and potassium iodide solution were applied in experiment might also affect the results. A variety of alternative methods have been employed to determine the amylose content of sorghum starch. The concanavalin A (ConA) method was employed in the studies reported by De Oliveira et al. (32) and Srichuwong et al. (33) to estimate AAC ranged from 11.50 to 22.75% and 24.6 to 25.8%, respectively. A low AAC range (0.25–27.90%) was investigated by Peiris et al. (34) using NIR spectroscopy. The higher AAC obtained in this study may be due to iodine-starch interactions (21). Additionally, differences in sorghum varieties and living environments may be responsible for the AAC gap.

**TABLE 2:** Apparent amylose content, moisture content, water solubility index, swelling power, relative crystallinity, degree of order, and degree of double helix of sorghum starch.

Sample	AAC (%)	MC (%)	WSI (%)	SP (g/g)	RC (%)	DO	DD
S1	9.45 ± 0.21^m^	8.9 ± 0.1^op^	49.8 ± 0.3^g^	35.1 ± 0.2^d^	31.6 ± 0.1^a^	0.762 ± 0.009^b^	1.514 ± 0.008^c^
S2	9.19 ± 0.27^m^	10.2 ± 0.1^kl^	26.4 ± 0.9^op^	40.6 ± 0.2^b^	31.2 ± 0.1^ab^	0.710 ± 0.009^efg^	1.433 ± 0.008^fg^
S3	8.17 ± 0.32^mn^	11.6 ± 0.1^gh^	34.8 ± 0.6^l^	45.4 ± 1.2^a^	30.6 ± 0.6^ab^	0.766 ± 0.010^b^	1.537 ± 0.009^b^
S4	7.42 ± 0.21^n^	9.0 ± 0.5^o^	24.3 ± 1.8^o^	45.7 ± 0.1^a^	32.4 ± 0.5^a^	0.797 ± 0.019^a^	1.579 ± 0.016^a^
S5	17.05 ± 0.53^j^	8.6 ± 0.4^pq^	46 ± 0.2i^j^	45.6 ± 0.7^a^	27.4 ± 0.5^cd^	0.689 ± 0.009^ghi^	1.427 ± 0.008^fghi^
S6	15.47 ± 0.53^k^	12.0 ± 0.1^ef^	44.6 ± 0.9^j^	38.0 ± 1.1^c^	28.2 ± 0.9^c^	0.713 ± 0.009^de^	1.405 ± 0.008^jkl^
S7	11.29 ± 0.33^l^	10.7 ± 0.2^ij^	29.1 ± 1.2^n^	35.6 ± 0.3^d^	29.2 ± 0.4^bc^	0.751 ± 0.005^bc^	1.512 ± 0.008^c^
S8	20.24 ± 0.59^i^	11.7 ± 0.3^fg^	49 ± 2.3^gh^	27.1 ± 0.8^fg^	26.0 ± 0.8^de^	0.690 ± 0.008^fgh^	1.395 ± 0.006^kl^
S9	22.18 ± 0.08^h^	11.2 ± 0.3^h^	32.3 ± 0.8^m^	27.6 ± 1.3^fg^	25.7 ± 3.0^defg^	0.676 ± 0.005^hij^	1.392 ± 0.004^l^
S10	27.01 ± 0.69^f^	9.7 ± 0.1^mn^	70.6 ± 0.8^a^	25.7 ± 0.1^h^	25.8 ± 1.7^def^	0.669 ± 0.010^ijk^	1.388 ± 0.008^lm^
S11	27.72 ± 0.74^ef^	12.1 ± 0.1^e^	43.8 ± 0.0^j^	26.4 ± 0.5^gh^	25.4 ± 0.5^defgh^	0.657 ± 0.009^jkl^	1.421 ± 0.008^ghij^
S12	28.85 ± 1.59^de^	8.7 ± 0.1^opq^	22.4 ± 0.5^p^	24.2 ± 0.9^i^	23.4 ± 0.3^ghijklm^	0.665 ± 0.008^jk^	1.392 ± 0.006^l^
S13	29.56 ± 0.80^d^	11.8 ± 0.3^efg^	47.3 ± 2.2^hi^	17.8 ± 0.2^mn^	24.3 ± 1.2^efghij^	0.655 ± 0.009^jkl^	1.394 ± 0.007^kl^
S14	25.17 ± 0.21^g^	12.7 ± 0.1^d^	36.7 ± 0.7^l^	20.8 ± 1.1^jk^	23.8 ± 0.3^efghijk^	0.711 ± 0.009^ef^	1.391 ± 0.007^l^
S15	26.93 ± 0.16^f^	13.1 ± 0.4^bc^	30.9 ± 0.6^n^	20.2 ± 1.1^jk^	24.6 ± 0.8^efghi^	0.706 ± 0.008^efg^	1.440 ± 0.007^ef^
S16	29.11 ± 0.27^de^	8.8 ± 0.2^opq^	44.2 ± 1.6^j^	34.7 ± 0.1^d^	21.5 ± 0.2^lmn^	0.626 ± 0.008^m^	1.366 ± 0.006^n^
S17	24.71 ± 1.06^g^	11.6 ± 0.3^gh^	43.8 ± 0.2^j^	39.9 ± 0.1^b^	28.5 ± 0.7^c^	0.698 ± 0.009^efg^	1.462 ± 0.007^d^
S18	29.30 ± 1.38^de^	13.5 ± 0.3^a^	40.7 ± 0.6^k^	19.6 ± 1.1^kl^	22.5 ± 0.1^ijklmn^	0.666 ± 0.009^jk^	1.372 ± 0.008^mn^
S19	27.83 ± 0.16^ef^	8.4 ± 0.3^q^	39.5 ± 0.2^k^	26.5 ± 0.4^gh^	22.1 ± 0.5^jklmn^	0.695 ± 0.009^efgh^	1.443 ± 0.007^ef^
S20	28.81 ± 0.05^de^	9.8 ± 0.1^lm^	41.5 ± 0.6^k^	30.3 ± 0.3^e^	24.3 ± 0.8^efghij^	0.731 ± 0.010^cd^	1.275 ± 0.001^p^
S21	34.30 ± 0.80^bc^	9.4 ± 0.2^n^	24.3 ± 0.4^op^	20.6 ± 0.9^jk^	23.6 ± 0.1^fghijklm^	0.602 ± 0.009n	1.357 ± 0.009^n^
S22	34.00 ± 0.16^bc^	9.7 ± 0.2^mn^	65.2 ± 2.1^b^	19.9 ± 0.2^kl^	23.3 ± 0.7^hijklm^	0.668 ± 0.008^jk^	1.415 ± 0.006^hij^
S23	33.25 ± 1.44^c^	10.8 ± 0.2^i^	45.5 ± 0.2i^j^	28.4 ± 0.3^f^	23.9 ± 2.6^efghij^	0.642 ± 0.009^lm^	1.431 ± 0.007^fgh^
S24	33.17 ± 1.01^c^	11.6 ± 0.1^fg^	69.9 ± 1.4^a^	21.6 ± 1.2^j^	21.4 ± 0.1^mn^	0.675 ± 0.008^hij^	1.388 ± 0.006^lm^
S25	33.55 ± 0.58^bc^	13.3 ± 0.1^ab^	60.2 ± 0.7^d^	20.5 ± 0.1^jk^	21.7 ± 0.4^klmn^	0.652 ± 0.009^kl^	1.293 ± 0.007^o^
S26	33.29 ± 0.21^c^	9.9 ± 0.1^lm^	54.6 ± 0.4^e^	14.8 ± 0.8^n^	25.2 ± 0.2^defgh^	0.664 ± 0.009^jk^	1.445 ± 0.008^ef^
S27	34.98 ± 0.58^ab^	10.4 ± 0.2^jk^	29.5 ± 0.6^n^	18.7 ± 1.2^lm^	20.5 ± 0.2^mn^	0.638 ± 0.008^lm^	1.410 ± 0.008^ijk^
S28	33.74 ± 0.32^bc^	11.5 ± 0.2^gh^	52.4 ± 0.3^f^	28.5 ± 0.2^f^	21.6 ± 0.1^klmn^	0.701 ± 0.009^efg^	1.433 ± 0.008^fg^
S29	36.44 ± 1.06^a^	12.8 ± 0.2^cd^	29.7 ± 0.6^n^	25.3 ± 1.4^hi^	23.7 ± 0.4^fghijkl^	0.734 ± 0.009^c^	1.427 ± 0.006^fghi^
S30	35.09 ± 0.64^ab^	12.1 ± 0.1^e^	63 ± 1.4^c^	17.3 ± 0.8^m^	25.2 ± 0.3^defgh^	0.694 ± 0.009^efgh^	1.455 ± 0.007^de^

Values in the same column with the different letters differ significantly (*p* < 0.05); AAC, apparent amylose content; MC, moisture content; WSI, water solubility index at 85°C; SP, swelling power at 85°C; RC, relative crystallinity; DO, degree of order; DD, degree of double helix.

The average value of AAC (25.58%) was much lower than that of wheat starch (38.6%) (35), but higher than 21.29% of quinoa starch (21) and 21.6% of rice starch (36). However, the average AAC value was lower in comparison to 31.1% of normal maize starch (37), which was obtained by the iodometric method.

### 3.2. Morphological properties

**FIGURE 1:**
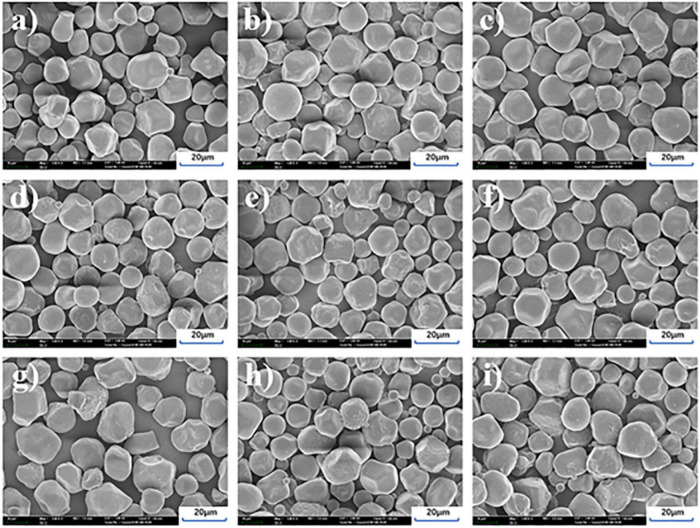
SEM images of different sorghum starch varieties. **(a–i)** Represent the sorghum starch samples S1 to S9.

No significant differences were observed in the SEM images of 30 different types of sorghum starches (Supplementary Figure 1). According to the different amylose content, nine kinds of starch were selected as shown in Figure 1. The starch granule morphology of sorghum exhibited irregular spherical or polygonal shapes. The presence of small pores and dents distributed on the surface of sorghum starch granules was obvious. The same finding was reported in previous study (38). These dents and pores extended from the outer surface of the starch granules to the internal part, which can affect the reaction of the granules with other substances, especially amylase (39). The average particle size of sorghum starch (14.5 μm) was calculated by ImageJ software, with the majority between 10 and 20 μm. The largest sorghum starch particles were approximately 24 μm while the smallest were approximately 2 μm. Sorghum starch granules were close to 5–25 μm of corn starch (40), larger than 1.21–1.95 μm of quinoa starch (41), but smaller than 22.51–52.79 μm of potato starch (42). Varying genes and growing environments resulted in different sizes and shapes of starch granules from distinct plant sources (43). Physical and chemical characteristics including swelling, pasting and enzyme sensitivity might be impacted by the size of the starch granules.

### 3.3. Swelling power (SP) and water solubility index (WSI)

**FIGURE 2:**
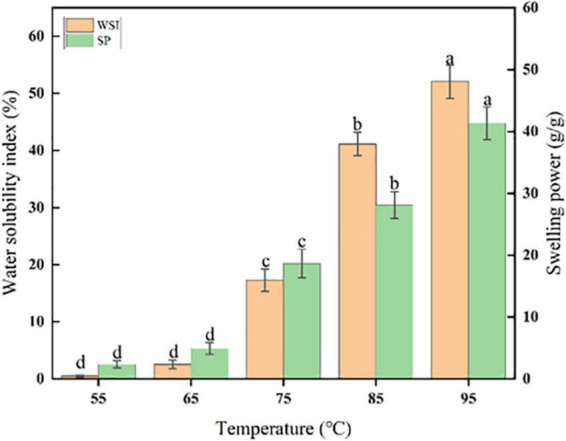
Average WSI and SP of sorghum starch. **(a–d)** Different letters indicate significant differences (*p* < 0.05).

According to Figure 2, the water solubility index (WSI) and swelling power (SP) of sorghum starch varied obviously as temperature increased. As temperature increased from 55 to 95 °C, the water solubility index and swelling force increased from 0.51 ± 0.07 to 52.16 ± 5.12% and 2.35 ± 0.59 to 41.35 ± 4.92 g/g, respectively. Sorghum starch had a greater water solubility index than that of quinoa starch (41) and rice starch (16), which could be because the particle size of sorghum starch is larger than that of quinoa and rice starch, resulting in a larger contact area between sorghum starch particles and water (11, 41).

**FIGURE 3:**
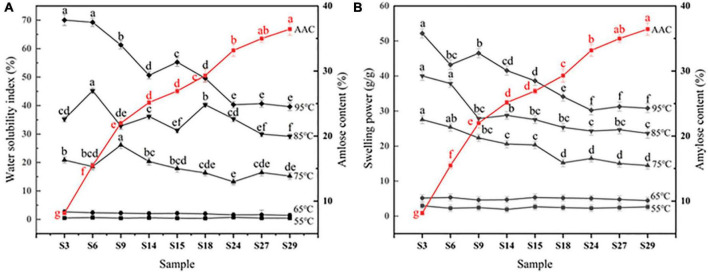
**(A)** Trends of water solubility index of sorghum starch with amylose content. **(B)** Trends of swelling power of sorghum starch with amylose content. S3–S29 are sorghum starch with gradually increasing amylose content. a–f: Different letters indicate significant differences (*p* < 0.05). There was no significant difference between different starches at 55 and 65 °C.

The relationship between WSI and AAC of sorghum starch would not be statistically significant (Figure 6). While the trends of WSI at 55, 65, and 75°C were relatively smooth (Figure 3A). WSI was found to be negatively correlated with AAC at 95°C, which could be attributed to the generation of amylose-lipid complex at high temperatures resulting in a denser structure (13, 44). The formation of this denser structure inhibited starch dissolution (45–47). Figure 3B depicted a negative correlation between SP and AAC of sorghum starch at 75, 85, and 95°C, which implied that amylose might limit starch granule swelling (48, 49). SP was significantly negatively correlated with AAC at 85°C (*r* = −0.80, *p* < 0.001) (Figure 6). Similar phenomenon was stated by Kong et al. (36) and Li et al. (21).

### 3.4. ATR-FTIR analysis

**FIGURE 4:**
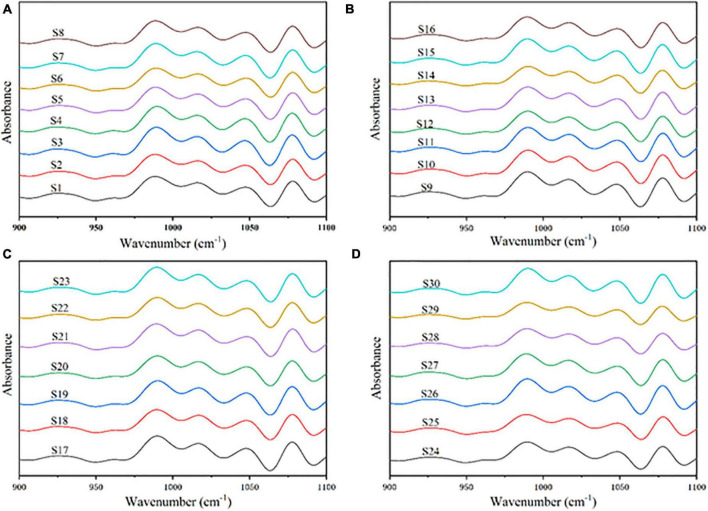
**(A)** Deconvoluted FTIR spectra from S1 to S8. **(B)** Deconvoluted FTIR spectra from S9 to S16. **(C)** Deconvoluted FTIR spectra from S17 to S23. **(D)** Deconvoluted FTIR spectra from S24 to S30.

Short-range ordered (crystalline) structures in starch have been reported to be sensitive to ATR-FTIR (50). The deconvoluted spectra of various sorghum starches as shown in Figure 4, they are largely caused by C–O and C–C stretching vibrations between 1,200 and 900 cm^–1^ (23, 51). This range is quite sensitive to the starch’s physical state. Absorbance at 1,045, 1,022, and 995 cm^–1^ presented starch’s conformational changes, and the ratios of 1,047 and 1,022 cm^–1^ were commonly employed to quantify the degree of order/crystallinity (52). The ratio of 1,047/1,022 cm^–1^ reflected the degree of order (DO) of sorghum starch crystalline area, and the ratio of 995/1,022 cm^–1^ is relative to the double helix (DD). Table 2 showed that the DO of sorghum starch ranged from 0.602 ± 0.009 in S21 to 0.797 ± 0.019 in S4, while the DD ranged from 1.275 ± 0.001 in S20 to 1.579 ± 0.016 in S4. The correlation plot in Figure 6 showed that DO and DD of sorghum starch were related to AAC. AAC exhibited negative correlation with both DO (*r* = −0.701, *p* < 0.001) and DD (*r* = −0.593, *p* < 0.001), which indicated that the degree of order and double helix of the sorghum starch were influenced by AAC. High amylose content was associated with lower DD and DO. The present of amylose could disrupt granular crystalline ordering and stabilization (53). The parameters of DD and DO were associated with SP at 85 °C. DO was positively correlated with SP (*r* = 0.585, *p* < 0.001) and DD was also positively correlated with SP (*r* = 0.489, *p* < 0.01). It showed that starch contained a high degree of order and double helix structure resulted in higher swelling power. The phenomenon corresponded to the findings of Du et al. (54) and Zhong et al. (53). The swelling of starch during heating was affected by its crystalline structure (16). DO and DD increased the strength of the crystalline structure, which might lead to starch granules swelling without breaking.

### 3.5. X-ray diffraction (XRD)

**FIGURE 5:**
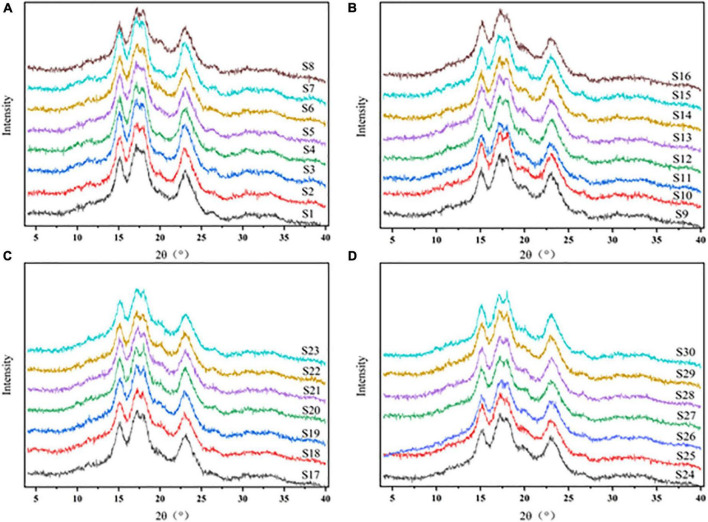
**(A)** X-ray diffraction from S1 to S8. **(B)** X-ray diffraction from S9 to S16. **(C)** X-ray diffraction from S17 to S23. **(D)** X-ray diffraction from S24 to S30.

As present in Figure 5, there is no significant difference was found on XRD patterns of sorghum starch. The characteristic peaks occurred at 15°, 17°, 18°, and 23° had the same pattern as normal grain starch (55). The XRD peak pattern of sorghum starch had diffraction peaks at 15° and 23° 2θ and constant double peaks at 17° and 18° 2θ (Figure 5). This was consistent with the peak pattern of other sorghum starches, which were all type A crystallization (56, 57). Although there is no difference in the XRD pattern of sorghum starch, starches revealed a great difference in crystallinity. Sorghum starch exhibited large characteristic peaks (Figure 5A), which reflected the large crystallinity. The relative crystallinity of sorghum starch ranged from 20.5 ± 0.2% in S27 to 32.4 ± 0.5% in S4, with significant differences (*p* < 0.05) between the thirty starches (Table 2). Pearson correlation analysis revealed that the RC of sorghum starch was highly correlated with AAC (*r* = −0.891, *p* < 0.001). Starch made up of an ordered crystalline region and an unordered amorphous region. Most of the noncrystalline and crystalline regions were made up of amylose and amylopectin, respectively (58). The relationship between AAC and RC also supported that amylopectin was the main contributor to the crystalline structure. The crystallinity of sorghum starch (18.86–23.87%) reported by Li et al. (59) was lower than that in this study. The main reason could attribute to limited number of sorghum varieties and a single sorghum source.

**FIGURE 6:**
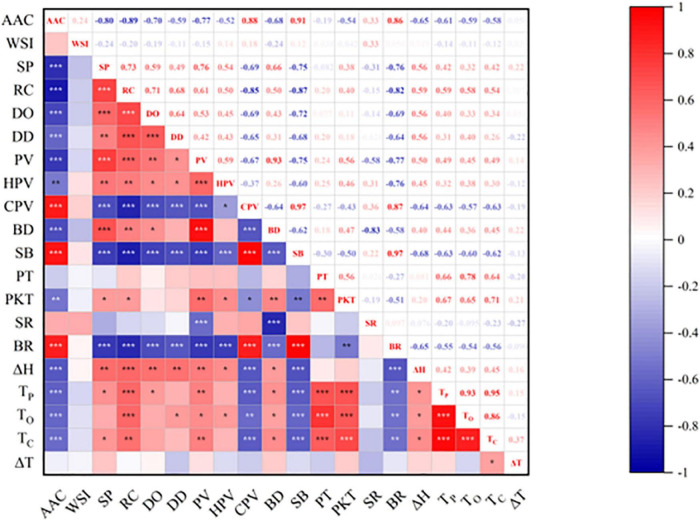
Pearson correlation coefficients among various physicochemical properties of sorghum starch. *p ≤ 0.05, **p ≤ 0.01, ***p ≤ 0.001.

Figure 6 showed that RC is significantly correlated with DO (*r* = 0.706, *p* < 0.001) and DD (*r* = 0.679, *p* < 0.001). The degree of short-range order and double helix of sorghum starch suggested to be related to the degree of crystallinity, which is the same with previous studies (60). This indicated that the starches had microcrystalline areas made up of ordered starch molecules (54).

### 3.6. Pasting properties

Great variation in pasting properties among 30 sorghum starch samples has been observed (Table 3). BD and SR revealed the resistance of paste to heat and shearing, while SB and BR represent the tendency of paste to retrograde and amylose to re-associate (36). S4 exhibited the largest PV (277.5 ± 2.3 RVU) and BD (170.0 ± 2.2 RVU), while S26 displayed the smallest PV (146.4 ± 27.9 RVU) and BD (74.6 ± 9.3 RVU). PV was related to the water absorption capacity or the degree of swelling of the particles (61). HPV ranged from 71.8 ± 6.6 RVU in S26 to 119.4 ± 2.7 RVU in S3. PKT ranged from 84.3 ± 0.5°C in S17 to 90.4 ± 0.1°C in S1. According to Figure 6, AAC was negatively related to PV, HPV, BD, and PKT. The correlation between PV and AAC was consistent with that between SP and AAC. These results indicated that the abilities of sorghum starch to combine with water and the degree of swelling were enhanced as AAC increased. BD was negatively correlated with AAC as reported in a previous study (17, 37). The PKT was decrease with the increase of AAC. It was because higher amount of the AAC, the faster starch paste reached PV. At a constant rate of warming for RVA, starch that reaches PV faster has smaller PKT. S29 showed the maximum CPV (244.5 ± 15.3 RVU), SB (160.7 ± 15.1 RVU), and SR (2.92 ± 0.18), while S4 revealed the minimum values of CPV (144.0 ± 1.6 RVU), SB (36.5 ± 1.7 RVU), and BR (1.34 ± 0.01). The CPV, SB and BR were found to be positively correlated with AAC, implying that the increase in AAC promoted retrogradation of starch. Due to the essentially linear structure, the amylose part of starch retrogrades more readily than amylopectin (36). The straight chain structure of amylose contributed to forming hydrogen bonds between molecules, which resulted in firm gels generation (62). The PT of sorghum starch was 77.3–83.3°C, which was higher than that of sorghum from India (17) and Algeria (18). Variables in starch pasting capabilities among cultivars may be due to genetic and growing environment differences.

**TABLE 3:** Pasting properties of sorghum starch.

Sample	PV (RVU)	HPV (RVU)	CPV (RVU)	BD (RVU)	SB (RVU)	PT (°C)	PKT (°C)	SR	BR
S1	261.0 ± 10.1^ab^	109.9 ± 0.8^ab^	149.6 ± 1.9^ij^	151.1 ± 9.4^bcd^	39.7 ± 1.2^i^	81.6 ± 0.0^b^	90.4 ± 0.1^a^	42.1 ± 1.3^cdefgh^	1.37 ± 0.01^k^
S2	260.2 ± 7.6^ab^	111.0 ± 1.7^ab^	164 ± 5.8^ij^	149.3 ± 5.9^bcde^	53.0 ± 9.1^hi^	83.3 ± 0.0^a^	90.3 ± 0.0^a^	42.7 ± 0.6^cdefgh^	1.48 ± 0.08^k^
S3	275.5 ± 5.2^a^	119.4 ± 2.7^a^	167.8 ± 9.9^ij^	156.1 ± 2.5^abc^	48.4 ± 7.1^i^	81.6 ± 0.1^b^	89.6 ± 0.1^ab^	43.3 ± 0.2^cdefgh^	1.41 ± 0.05^k^
S4	277.5 ± 2.3^a^	107.5 ± 0.1^bc^	144.0 ± 1.6^j^	170.0 ± 2.2^a^	36.5 ± 1.7^i^	79.9 ± 1.2^defgh^	85.5 ± 0.0^hi^	38.7 ± 0.3^hijk^	1.34 ± 0.01^k^
S5	247.9 ± 3.6^bc^	83.9 ± 1.9^fghi^	172.4 ± 4.1^hi^	164.1 ± 1.8^ab^	88.6 ± 2.2^g^	80.0 ± 1.1^cdefg^	87.5 ± 0.6^de^	33.8 ± 0.3^lm^	2.06 ± 0.01^ij^
S6	248.6 ± 3.3^bc^	86.9 ± 3.5^efghi^	162.6 ± 3.1^ij^	161.8 ± 0.1^ab^	75.8 ± 6.5^gh^	80.8 ± 0.0^bcde^	88.0 ± 0.0^cd^	34.9 ± 0.9^jklm^	1.88 ± 0.11^j^
S7	261.1 ± 0.8^ab^	96.1 ± 0.9^de^	151.2 ± 1.0^ij^	165.0 ± 0.1^ab^	55.2 ± 0.1^hi^	80.9 ± 0.0^bcd^	89.2 ± 0.1^b^	36.8 ± 0.2^ijklm^	1.58 ± 0.01^k^
S8	235.3 ± 1.9^cd^	79.9 ± 0.4^hij^	201.2 ± 1.3^fg^	155.4 ± 1.5^abc^	121.3 ± 1.7^def^	80.5 ± 0.3^bcdef^	87.5 ± 0.1^de^	34.0 ± 0.1^klm^	2.52 ± 0.03^bcdefgh^
S9	235.4 ± 0.2^cd^	77.9 ± 1.5^ij^	210.9 ± 0.6^defg^	157.5 ± 1.3^ab^	133.1 ± 0.9^bcdef^	79.6 ± 0.1^efgh^	87.3 ± 0.1^def^	33.1 ± 0.6^m^	2.71 ± 0.04^abcde^
S10	192.0 ± 9.8^ij^	102.8 ± 8.1^bcd^	244.4 ± 5.7^a^	89.2 ± 8.3^o^	147.6 ± 3.4^abcd^	79.5 ± 1.9^fghi^	87.6 ± 0.5^de^	53.4 ± 6.7^a^	2.47 ± 0.29^cdefgh^
S11	201.1 ± 4.6^hij^	89.1 ± 1.6^efghi^	218.9 ± 0.5^bcdefg^	112.0 ± 6.2^lmn^	129.8 ± 1.1^cdef^	78.8 ± 0.6^hi^	86.4 ± 0.0^fgh^	44.3 ± 1.8^cdefg^	2.46 ± 0.04^cdefgh^
S12	210.1 ± 9.3^fgh^	96.8 ± 8.6^cde^	240.0 ± 16.5^abcd^	113.3 ± 2.7^klmn^	143.2 ± 7.9^abcde^	80.8 ± 0.1^bcde^	87.1 ± 0.0^def^	46.0 ± 1.6^bcd^	2.49 ± 0.05^bcdefgh^
S13	214.4 ± 0.8^efgh^	88.5 ± 6.6^efghi^	237.5 ± 10.6^abcd^	126.0 ± 7.4^fghijkl^	149.1 ± 12.2^abc^	80.8 ± 0.1^bcde^	86.4 ± 0.1^fgh^	41.3 ± 3.2^defghi^	2.7 ± 0.47^abcdef^
S14	188.5 ± 10.3^j^	82.2 ± 3.0^ghij^	232.1 ± 9.7^abcde^	106.4 ± 7.4^mn^	149.9 ± 10.7^abc^	80.7 ± 0.0^bcde^	85.6 ± 0.0^hi^	43.6 ± 0.8^cdefgh^	2.83 ± 0.15^ab^
S15	222.6 ± 2.8^defg^	87.4 ± 2.1^efghi^	220.6 ± 5.4^bcdefg^	135.3 ± 0.8^efgh^	133.3 ± 3.3^bcdef^	80.8 ± 0.1^bcde^	86.0 ± 0.6^ghi^	39.3 ± 0.4^ghij^	2.53 ± 0.01^bcdefgh^
S16	232.1 ± 6.7^cde^	92.7 ± 1.6^defg^	241.3 ± 11.4^abc^	139.5 ± 8.3^defg^	148.6 ± 6.0^abc^	79.2 ± 0.1^ghi^	88.0 ± 1.1^cd^	39.9 ± 1.8^fghij^	2.61 ± 0.16^abcdefg^
S17	214.6 ± 0.2^efgh^	96.2 ± 0.4^de^	220.3 ± 7.8^bcdefg^	118.4 ± 0.6^ijklmn^	124.1 ± 7.4^cdef^	77.4 ± 0.1^i^	84.3 ± 0.5^i^	44.8 ± 0.2^bcdef^	2.29 ± 0.07^ghi^
S18	221.7 ± 4.0^defg^	97.7 ± 0.3^cde^	218.9 ± 11.4^bcdefg^	124.0 ± 3.7^ghijkl^	121.1 ± 10.7^ef^	80.8 ± 0.0^bcd^	87.2 ± 0.0^def^	44.1 ± 0.7^cdefg^	2.24 ± 0.13^hi^
S19	212 ± 7.1^fgh^	89.5 ± 0.7^efgh^	204.5 ± 4.9^efg^	122.5 ± 6.4^hijklm^	115.0 ± 5.6^f^	78.8 ± 0.6^hi^	85.6 ± 1.1^hi^	42.2 ± 1.1^cdefgh^	2.29 ± 0.08^ghi^
S20	211.9 ± 1.3^fgh^	92.1 ± 5.7^defg^	221.5 ± 7.8^abcdefg^	119.9 ± 7.0^hijklmn^	129.4 ± 6.2^cdef^	77.3 ± 0.0^i^	85.6 ± 0.0^hi^	43.5 ± 2.9^cdefgh^	2.41 ± 0.02^defgh^
S21	215.4 ± 3.5^efgh^	89.5 ± 0.2^efgh^	234.6 ± 6.7^abcd^	126.0 ± 3.8^fghijkl^	145.2 ± 6.9^abcde^	81.2 ± 0.5^bcd^	88.8 ± 0.0^bc^	41.5 ± 0.8^defghi^	2.63 ± 0.08^abcdefg^
S22	216.6 ± 2.8^efgh^	83.4 ± 0.8^fghi^	232.1 ± 5.3^abcde^	133.2 ± 3.5^fghi^	148.7 ± 4.5^abc^	80.9 ± 0.0^bcd^	87.2 ± 0.0^def^	38.5 ± 0.8^hijkl^	2.79 ± 0.04^abc^
S23	228 ± 2.8^def^	97.1 ± 5.1^cde^	228.6 ± 3.8^abcdef^	130.9 ± 7.9^fghij^	131.6 ± 8.8^cdef^	80.8 ± 0.0^bcde^	86.4 ± 0.1^fgh^	42.6 ± 2.8^cdefgh^	2.36 ± 0.16^fghi^
S24	227.9 ± 3.0^def^	98.5 ± 5.0^cde^	233.1 ± 13.6^abcde^	129.4 ± 2.0^fghijk^	134.7 ± 8.6^abcdef^	80.8 ± 0.0^bcd^	86.0 ± 0.5^ghi^	43.2 ± 1.6^cdefgh^	2.37 ± 0.02^efghi^
S25	231.6 ± 4.2^cde^	90.7 ± 3.3^efgh^	229.7 ± 11.1^abcdef^	140.9 ± 7.5^cdef^	139.0 ± 6.4^abcdef^	81.2 ± 0.6^bcd^	86.4 ± 0.1^fgh^	39.2 ± 2.1^ghij^	2.54 ± 0.21^bcdefgh^
S26	146.4 ± 27.9^k^	71.8 ± 6.6^j^	196.7 ± 37.2^gh^	74.6 ± 9.3^p^	124.9 ± 10.5^cdef^	81.2 ± 0.5^bc^	85.5 ± 0.0^hi^	49.5 ± 4.9^ab^	2.73 ± 0.27^abcd^
S27	206.9 ± 1.2^ghi^	84.3 ± 4.9^fghi^	242.6 ± 0.8^abc^	122.5 ± 3.8^hijklm^	158.3 ± 4.2^ab^	80.8 ± 0.1^bcde^	86.9 ± 0.6^efg^	40.8 ± 2.2^efghi^	2.88 ± 0.16^a^
S28	212.9 ± 5.8^fgh^	97.7 ± 4.5^cde^	236.1 ± 8.2^abcd^	115.2 ± 1.3^jklmn^	138.4 ± 3.7^abcdef^	80.8 ± 0.0^bcd^	87.6 ± 0.6^de^	45.9 ± 0.9^bcde^	2.42 ± 0.03^defgh^
S29	212.8 ± 8.9^fgh^	83.9 ± 0.2^fghi^	244.5 ± 15.3^a^	129.0 ± 9.6^fghijk^	160.7 ± 15.1^a^	80.8 ± 0.1^bcde^	86.0 ± 0.6^ghi^	39.5 ± 2.5^ghij^	2.92 ± 0.18^a^
S30	199.6 ± 7.2^hij^	93.8 ± 1.4^def^	213.9 ± 0.5^cdefg^	105.8 ± 8.6^n^	120.0 ± 1.0^ef^	80.7 ± 0.0^bcde^	85.2 ± 0.5^i^	47.1 ± 2.4^bc^	2.28 ± 0.03^ghi^

Values in the same column with the different letters differ significantly (*p* < 0.05); PT, pasting temperature; PV, peak viscosity; PKT, peak temperature; HPV, hot paste viscosity; CPV, cold paste viscosity; BD, breakdown (PV - HPV); SB, setback (CPV - HPV); SR, stability ratio (100 × HPV/PV); BR, setback ratio (CPV/HPV).

### 3.7. Thermal properties

The gelatinization temperatures and enthalpy of the endothermic peaks were presented in Table 4. The Δ*H* value, *T*o, *T*p, *T*c, and Δ*T* (*T*c-*T*o) ranged from 7.87 ± 0.27 to 12.92 ± 0.42 J/g, 67.3 ± 0.4 to 75.8 ± 0.3°C, 73.5 ± 0.1 to 80.8 ± 0.1°C, 78.5 ± 0.2 to 85.7 ± 0.1°C, and 8.3 ± 0.3 to 12.4 ± 0.1°C, respectively. The thermal properties were similar with the literature reported by Singh et al. (17). The Δ*H* value is inconsistent with research data in some of the literature (15, 63). The differences in amylose content, starch granule size and the presence of minor components (such as proteins and lipids) may explain the diversity of sorghum starch in gelatinization behaviors (64). The *T*o, *T*p, and *T*c values were similar to amaranth starch (65) and larger than quinoa starch (41). As exhibited in Table 4, S1, S2, and S3 have larger *T*o, *T*p, and *T*c values which indicates the higher stability of these starches. The Δ*H* of sorghum starch is less than that of rice starch (16, 36). The Δ*T* value indicated the stability and uniformity of the crystalline region in starch granule (66). A high Δ*T* indicates that the crystalline regions of the starch granules contained many different crystallites (67). The Δ*T* of sorghum starch was less than that of sago starch (54) and quinoa starch (41).

**TABLE 4:** Thermal properties of sorghum starch.

Sample	Δ*H* (J/g)	*T*_*O*_ (°C)	*T*_*P*_ (°C)	*T*_*C*_ (°C)	Δ *T* (°C)
S1	10.4 ± 0.08^bcde^	74.3 ± 0.5^b^	78.7 ± 0.5^b^	83.6 ± 0.8^b^	9.4 ± 0.4^ijkl^
S2	9.77 ± 0.51^cdefgh^	75.8 ± 0.3^a^	80.8 ± 0.1^a^	85.7 ± 0.1^a^	9.9 ± 0.4^ghijk^
S3	12.40 ± 0.23^a^	73.2 ± 0.3^c^	78.7 ± 0.2^b^	83.6 ± 0.2^b^	10.4 ± 0.5^defg^
S4	12.92 ± 0.42^a^	71.8 ± 0.1^def^	76.5 ± 0.1^efg^	81.3 ± 0.2^efgh^	9.5 ± 0.1^hijkl^
S5	8.78 ± 0.30^hijk^	71.5 ± 0.4^defg^	77.2 ± 0.7^de^	82.3 ± 0.4^cd^	10.9 ± 0.8^cde^
S6	11.00 ± 0.44^b^	71.8 ± 0.6^def^	77.2 ± 0.1^def^	83.0 ± 0.2^bc^	11.2 ± 0.8^bcd^
S7	10.52 ± 0.10^bcd^	72.5 ± 0.2^cd^	78.2 ± 0.1^bc^	83.3 ± 0.1^b^	10.8 ± 0.1^cde^
S8	10.35 ± 0.16^bcde^	71.5 ± 0.2^defg^	76.4 ± 0.3^fg^	82.2 ± 0.1^cde^	10.8 ± 0.1^cdef^
S9	10.23 ± 0.05^bcdefg^	70.8 ± 0.2^fghi^	76.3 ± 0.3^fg^	81.7 ± 0.1^defg^	11.0 ± 0.4^cde^
S10	9.99 ± 0.06^bcdefg^	71.6 ± 0.3^defg^	76.4 ± 0.5^fg^	81.1 ± 0.7^fgh^	9.5 ± 0.4^hijkl^
S11	8.21 ± 0.13^k^	68.4 ± 0.1^m^	73.6 ± 0.1^k^	78.6 ± 0.1^lm^	10.2 ± 0.0^efgh^
S12	8.32 ± 0.14^jk^	70.7 ± 0.1^fghij^	74.7 ± 0.2^hi^	79.2 ± 0.4^klm^	8.5 ± 0.2^mn^
S13	8.39 ± 0.17^jk^	69.9 ± 0.1^hijk^	74.0 ± 0.1^ijk^	78.2 ± 0.0^m^	8.4 ± 0.1^mn^
S14	9.26 ± 0.46^ghij^	71.1 ± 0.1^efg^	75.1 ± 0.1^h^	79.4 ± 0.1^jkl^	8.3 ± 0.3^mn^
S15	9.5 ± 0.13^defghi^	69.7 ± 0.1^ijkl^	74.3 ± 0.2^hijk^	79.1 ± 0.4^klm^	9.5 ± 0.5^hijkl^
S16	9.43 ± 1.79^efghi^	68.6 ± 0.1^lm^	73.6 ± 0.0^k^	78.5 ± 0.2^lm^	9.9 ± 0.1^ghijk^
S17	10.33 ± 0.18^bcdef^	69.0 ± 0.4^klm^	73.7 ± 0.2^k^	78.5 ± 0.1^lm^	9.5 ± 0.3^hijkl^
S18	9.86 ± 0.57^cdefg^	69.9 ± 1.1^hijk^	75.0 ± 0.2^h^	80.2 ± 0.5^ij^	10.3 ± 0.6^efgh^
S19	9.63 ± 0.28^cdefghi^	68.2 ± 0.1^mn^	73.7 ± 0.1^jk^	78.4 ± 0.1^lm^	10.3 ± 0.1^efgh^
S20	9.28 ± 0.08^ghij^	67.3 ± 0.4^n^	74.5 ± 0.7^hij^	79.7 ± 0.3^jk^	12.4 ± 0.1^a^
S21	8.69 ± 0.09^ijk^	70.8 ± 0.6^fgh^	76.0 ± 0.3^g^	82.3 ± 0.8^cd^	11.5 ± 0.2^bc^
S22	8.33 ± 0.10^jk^	69.7 ± 1.6^hijkl^	74.7 ± 1.2^hi^	79.0 ± 1.3^klm^	9.3 ± 0.3^jkl^
S23	10.15 ± 0.11^bcdefg^	70.6 ± 0.1^ghij^	75.0 ± 0.0^h^	79.7 ± 0.1^jk^	9.1 ± 0.1^klm^
S24	9.31 ± 0.08^fghij^	71.4 ± 0.3^defg^	75.8 ± 0.3^g^	80.4 ± 0.1^hij^	9.0 ± 0.2^lmn^
S25	9.62 ± 0.42^cdefghi^	71.7 ± 0.4^defg^	76.9 ± 0.1^def^	81.9 ± 0.6^defg^	10.2 ± 0.2^efghi^
S26	10.62 ± 0.45^bc^	72.1 ± 1.0^de^	77.5 ± 0.4^cd^	82.1 ± 0.5^cdef^	10.0 ± 0.5^fghij^
S27	9.43 ± 0.16^efghi^	71.4 ± 0.1^defg^	76.3 ± 0.4^fg^	82.0 ± 0.2^defg^	10.6 ± 0.1^defg^
S28	10.17 ± 0.38^bcdefg^	69.6 ± 0.3^jkl^	74.8 ± 0.1^hi^	81.4 ± 0.0^defg^	11.8 ± 0.3^ab^
S29	7.87 ± 0.27^k^	71.2 ± 0.1^efg^	77.1 ± 0.1^def^	81.0 ± 0.1^ghi^	9.9 ± 0.1^ghijk^
S30	8.67 ± 0.07^ijk^	69.7 ± 0.1^hijkl^	73.5 ± 0.1^k^	79.6 ± 0.1^jk^	9.9 ± 0.2^ghijk^

Values in the same column with the different letters differ significantly (*p* < 0.05); ΔH, gelatinization enthalpy; *T_O_*, gelatinization onset temperature; *T_P_*, gelatinization peak temperature; *T_C_*, gelatinization conclusion temperature; Δ*T*, gelatinization temperature range (Tc – To).

The *T*o, *T*p, *T*c, and Δ*H* were negatively correlated with AAC (Figure 6). Similar finding was investigated in rice starch (36), and the result was opposite to that of amaranth starch (65). During the pasting process, Δ*H* represents the energy needed for unwinding and melting of the double helix structure (68). RC was positively correlated with *T*o, *T*p, *T*c, and Δ*H*. The results indicated dissociation of the double helix and the reduction of the order in the high crystalline starch crystals region required more heat and higher temperature. As shown in Figure 6, Δ*H* is positively correlated with RC, DO, and DD, which indicated that the crystallinity and degree of double helix of sorghum starch affects the enthalpy of gelatinization. PT was found to be positively related to *T*o, *T*p, and *T*c (Table 4). Therefore, the thermal properties could be an alternative evaluation method when it came to compare the magnitude of starch pasting temperature.

### 3.8. Principal component analysis (PCA)

Principal component analysis (PCA) plots can display the interrelationships between starch structural properties and the differences and similarities among diverse starches. (69, 70). The eigenvalues of the 17 principal components could explain all the variances. The contributions of various structural parameters were represented by the lengths of loading vectors (71). The loading plot in PCA revealed the relationship between measured attribute parameters. The properties of positive correlation were expressed as the lines that are close to each other on the graph, while the properties of negative correlation have lines that go in opposing directions. The first principal component explained 50.8% of total variance, which mainly contributed by AAC, RC, SP, PV, CPV, SB, and BR. AAC, RC, SP, PV, CPV, SB, and BR were distributed along PC1 and these properties were influenced by the AAC. The second principal component explained 12.3% of total variance, which was mainly contributed by DO, PT, PKT, *T*o, *T*p, and *T*c.

**FIGURE 7:**
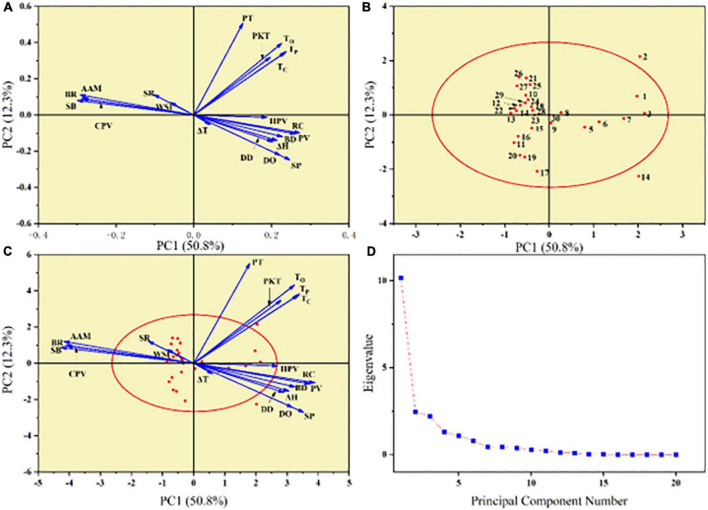
Principle component analysis (PCA) describing the variations in physicochemical properties of sorghum starch. **(A)** Loading plot of PC1-PC2. **(B)** Score plot of PC1-PC2. **(C)** Combination of loading and score plots of PC1-PC2. **(D)** Scree plot for 30 sorghum starch samples.

According to Figure 7A, AAC, SB, BR, and CPV were located on the left side of the PCA plot and clustered together. And RC, PV, and BD were located on the right side of the PCA plot and were also clustered together. This indicated that there was a strong correlation between these properties, which is agree with the correlation analysis in Figure 6.

Figure 7B showed the score plot of 30 different sorghum starches. The degree of variance or similarity in physicochemical qualities is related to the distance between the positions of any two starches on the score plot. The short distance of sorghum starch in score plot represented for similar properties (21). As seen in Figure 7B, the distribution of starch species was correlated with AAC. The small AAC variety was located on the right side of the PCA plot and the large AAC on the left. Except S2 and S14, starches were distributed near the PC1 and PC2 axes. The deviations of S2 and S14 may be due to differences in genes and growth environment. Some starches located in the region surrounded by the negative direction of PC1 and the positive direction of PC2 were close in distance, which indicated that they have high similarity in physicochemical properties.

## 4. Conclusion

In summary, starches isolated from 30 varieties of sorghum exhibited significant differences in physicochemical properties. In this study, the morphological properties, apparent amylose content, thermal properties, swelling properties, crystalline structure and pasting properties of starch were synthetical investigated. The correlation analysis revealed that the thermal properties, pasting properties, crystalline properties, water solubility index and swelling power of sorghum starch were all related to the AAC. AAC prevented the expansion of starch granules and decreased the thermal characteristics (*T*o, *T*p, *T*c, and Δ*H*). The greater AAC existed in sorghum starch, resulting in the lower the PV and BD. In addition, high level of AAC in sorghum starch increased the HPV and SB. The RC, DO, and DD of starch were positively correlated with AAC. The thermal properties, pasting properties and swelling power have a strong correlation to RC, DO, and DD. Principal component analysis (PCA) was conducted for 20 variables, based on the differences between 30 types of sorghum starch. The PCA1 and the PCA2 were the two most significant components, which explained most of the variance. The results of this study can be utilized to direct the choice and breeding of sorghum cultivars as well as the application of sorghum starch.

## Data availability statement

The original contributions presented in this study are included in the article/Supplementary material, further inquiries can be directed to the corresponding author.

## Author contributions

SY: investigation, methodology, and writing—original draft. ZaL and BW: supervision and project administration. TL, ZyL, and NZ: investigation and formal analysis. BC: conceptualization, resources, supervision, and funding acquisition. All authors contributed to the article and approved the submitted version.
