# Supplementary figures and images for: Correlation analysis on physicochemical and structural properties of sorghum starch
Source: Front Nutr. 2023 Jan 10;9:1101868. doi: 10.3389/fnut.2022.1101868 (PMC9873550; doi:10.3389/fnut.2022.1101868)

[**Supplemental**](javascript:;) [**file**](javascript:;)


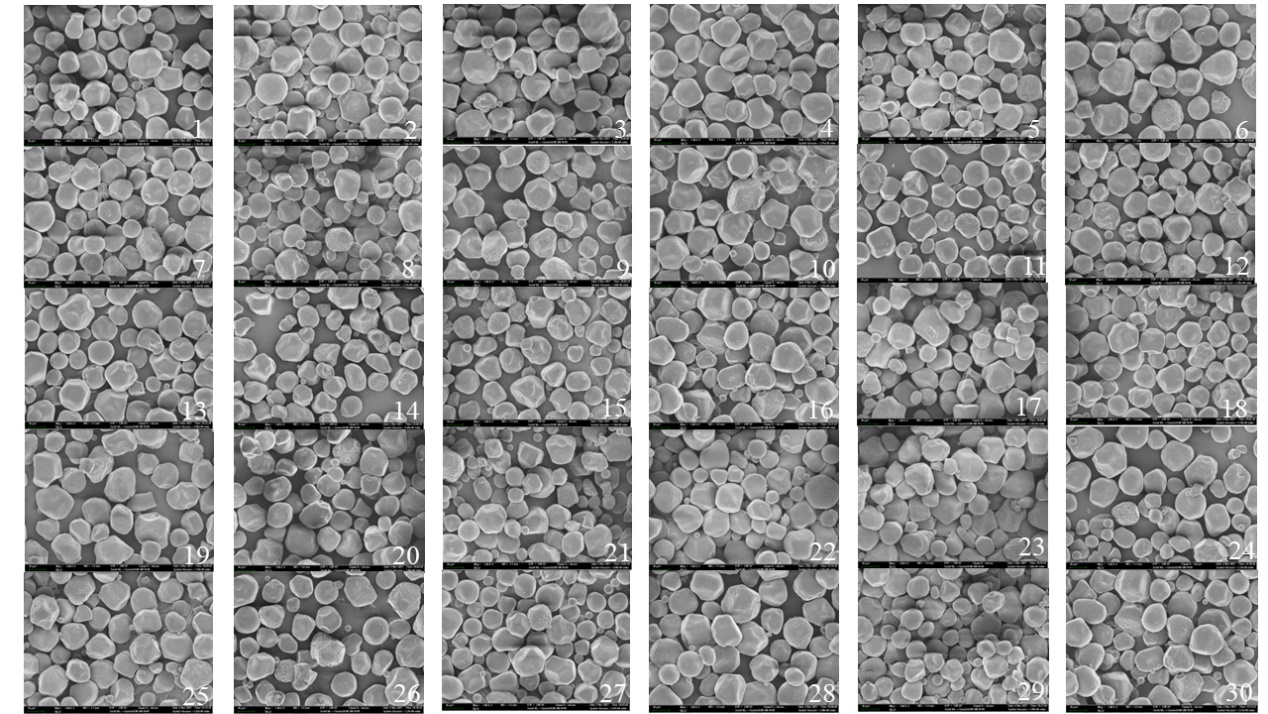


**Fig. s1**. SEM images of 30 kinds of sorghum starch.

Supplement: Supplementary file 1 [file Table_2.DOCX]
